# Abstracts and Gleanings

**Published:** 1885-03-20

**Authors:** 


### ABSTRACTS AND GLEANINGS.

**Pneumonia Treated with Jaborandi.**—Dr. J. W. Brown (Journal American Medical Association) gives a plan of treating pneumonia with jaborandi as described in the following illustrative cases:

CASE 1.—Mrs. S., aged 80, merchant's wife. Had had pneumonia, according to her own account, several times before at Del Norte, about seven thousand feet above sea-level. Had probably had pneumonia before, because she detailed the symptoms of pneumonia of her own accord, viz.: A chill, with a sense of shortness of breath, followed by pain in the chest, cough, bloody expectoration, fever, prostration and three or four weeks sickness, she being at the point of death part of the time, and all the while under the care of a regular practitioner. About five years ago was called to see her. Found her in a rigor which was very marked. Her face was cyanosed, extremities cold. She was shaking from head to foot and her teeth were chattering as though she had an ague, and she complained of a shortness of breath. She said: "I am going to have pneumonia: I recognize it, and I am afraid of the result." She was then living 9,400 feet above the sea: Ordered her put to bed immediately, and prescribed:

℞ Ext. jaborandi fl.................................ʒ iv

Spts. ammon. aromat...............................*m* xl

Syrupi simplicis...................................ʒ iv.

M. S. One teaspoonful every two or three hours to induce profuse perspiration.

She was put to bed, covered heavily with blankets, surrounded with hot irons wrapped in wet cloths, the irons being placed close to her body and the mixture administered as directed. In about twenty minutes she commenced to perspire profusely, and the sweating was maintained at its highest pitch for about six hours. Her bedding and clothing were then changed, her body wiped dry and fifteen drops of the mixture were administered sufficiently often during the night to keep her skin moist. The next morning she got up at her usual time and resumed her household duties. During the day she expectorated a small quantity of pneumonic sputum and complained of a slight feeling of soreness in the lower lobe of the right lung, but recovery was almost immediate. She did not go to bed again on account of this sickness and she had no further medication on that account.

CASE 2.—J. B., a miner, aged 54. Lived in a miner's cabin about four miles out of town, and at an elevation of about 10,000 feet. On inquiry, found that he had the symptoms of commencing pneumonia. Prescribed the mixture of the above prescription, and gave directions as in the preceding case. The next day the brother reported the patient better in some respects, but worse in others. He said the patient had retention of urine. Went up to see him and found him on his knees in bed vainly trying to void.

his urine into a tin cup. Introduced a catheter and found the bladder almost completely empty. Ordered cold water to be drank in plentiful quantity, the patient having abstained from drinking water for fear of aggravating the supposed retention of urine. He expectorated pneumonic sputa for two or three days, but within a week he resumed his work. In this case the sweating was so extreme that the skin had temporarily almost entirely usurped the function of the kidneys in the elimination of water, and the small quantity of urine in the bladder—a few drops only—was very acrid and irritating. The kidneys resumed their function in a few hours.

CASE 3.—J. K., aged 24, a miner. Lived at Ophir, thirteen miles away, across a mountain range, and at an elevation of 10,000 feet. He was taken with a chill at four o'clock in the morning. I arrived at his bedside at half-past four o'clock in the afternoon, so that the disease had a start of twelve hours and a half. From description given by messenger, a diagnosis of pneumonia was made out. The patient was in bed. Face was dull and leaden in color; he had fever, cough, pain in the right lung, and a sense of shortness of breath, and the floor near the head of the bed was covered with bloody expectoration. The floor was spattered with pneumonic sputa all over an area three or four feet in extent in all directions. Physical examination showed a dulluess in the lower part ot right lung, with subcrepitant rales. This was a case of plastic pneumonia beyond the shadow of a doubt, and it had a start of twelve and a half hours. He was treated in the same manner as the two preceding cases. In a quarter of an hour beads of sweat stood out on his forehead, and the patient was informed of the certainty of a speedy recovery.

As the sweating progressed, he experienced proportionate relief. The sweating was continued at the highest pitch for nearly eight hours. Ordered a diminished dose of the mixture, continued for two or three days, gave general directions for his further management, and dismissed him with orders to report, if necessary. In a week he resumed his work, having made a complete recovery.

CASE 4.—J. C., saloon-keeper, aged 24. Had had pneumonia two years previously at Leadville, and was confined to his bed three weeks, being very dangerously ill, according to his own statement, most of the time. He spoke very flatteringly of the physician in charge, on account of the good care rendered, and expressed himself as very lucky that he finally recovered, the prevalence of the disease and the rate of mortality being exceedingly great at that time at Leadville. Was called to see him on a Sunday afternoon, at half-past three. He had been taken with a chill at seven o'clock in the morning of the same day, so that the disease had a start of eight hours and a half. His pulse was full and bounding; expectoration profuse and typically pneumonic; there were fever, a dull leaden face, pain in the right lung, cough, and a sense of a want of breath. Examination gave the physical signs of pneumonia. He said it was the same thing he had at Leadville. He was treated in the same way as the preceding cases, with the same exceedingly profuse diaphoresis as a result, and the same relief. He remained in bed till the following Thursday morning, when he got up at the usual time and resumed his regular occupation. He was dismissed on Thursday morning without further treatment, and two days after was apparently well.

Other cases may be given, and some who have walked the street within two days expectorating pneumonic sputa, after being subjected to the jaborandi sweatbath ordeal, recovered without relapse or further inconvenience, convalescence continuing to perfect recovery in a few days thereafter.

**The Medical Uses of Iodine.**—J. J. Berry, M.D., of South Norwalk, Conn., says:

The highly satisfactory results which I have been able to secure by the use of this drug leads me to offer some clinical facts regarding its action. During the past year I have made use of the remedy quite extensively, having prescribed it, during this period, perhaps over a hundred times, and in diseases which had absolutely no syphilitic history. In a large proportion of cases the results have been highly satisfactory. Iodine and its compounds have been known to the profession for many years, but their employment has been limited to chronic affections. For this reason I desire to call the attention of the readers of your journal to its curative effects in acute diseases. First in the list stands malarial poisoning. From my own observations, as well as from the experience of others, I am convinced that in this affection it ranks little below the cinchona salts in efficacy. Since the first of December I have prescribed iodine in the various forms of acute malaria twenty-six times, and have experienced but six failures. That the success attained in this series of cases was not due, in a measure, to the cathartics used I am not prepared to state. Yet the same thing is to be taken into account when quinine is employed. The preparation with which I have had the best success is the compound tincture of iodine, the formula of which is :

| | | |
|---|---|---|
| ℞. | Iodine .................................... | ʒ ii, |
| | Potass. iod ................................. | ʒ iv, |
| | Spts. vini rect............................... | ℥ viii. |
| M. | | |

The amount of iodide of potash in each dose is too insignificant to possess much curative value, yet it fills an important place by rendering the iodine more soluble and possibly more acceptable to the stomach. The dose to be employed varies with the results we wish to obtain. Ordinarily 10 to 20 drops every three or four hours is sufficient. Given well diluted and at the proper time it seldom disturbs the stomach. It is in other respects much more acceptable than quinine. In the more chronic forms of malarial poisoning I am in the habit of giving larger doses of the drug, less frequently repeated, and with it arsenious acid in gradually increasing doses. These two drugs, used in conjunction with remedies which have a special action upon the liver, have seldom failed, in my experience, to effect a cure. I would here call attention to the value of Donovan's solution as an anti-malarial remedy.

In gastric irritation, from malarial as well as other causes, often repeated doses of one or two drops are exceedingly valuable. In the vomiting of pregnancy and chronic disease it proves fully as efficacious as other remedies. Its antiseptic properties render it valuable in typhoid fever, diphtheria and allied diseases of a septic nature, for the fact is clearly established that certain antiseptics have a decided influence upon the growth and increase of disease germs in the human economy. My experience here has been limited to a few cases, all of which terminated favorably. The observations of others, which have been more extended, show that typhoid fever patients, who are treated with iodine, have, usually, no high exacerbations of temperature, and no symptoms of intestinal ulceration—views which I have reason to support.

Iodine was advocated several years ago by Schwartz as a remedy for pneumonia. He claimed that, given in the initial stages, it cut short the disease. I have no doubt of its efficacy in acute congestions of the respiratory organs. In cases of acute bronchitis and of intense pulmonary engorgement, I have received very satisfactory results from moderately large doses frequently repeated. Recent experiments have shown that it is the most powerful antisuppurative remedy which we possess. I can bear witness to its value in acute inflammation of the tonsils, both in preventing the formation of pus and in controlling the congestion of these parts.

My experience in the use of iodine is not sufficient to render my deductions of any great value. My object is to induce others to carefully study the effects of this remedy and give the results of their experience.—*New England Medical Monthly.*

**Two New Methods for the Recognition of Albumen and Sugar in the Urine.**—Dr. Baas publishes the following procedure for the recognition of albumen and sugar in the urine by means of test-papers.

### METHOD FOR THE DETECTION OF ALBUMEN.

One strip of paper is soaked in concentrated citric acid and dried, and may be stained red with litmus to distinguish it from the other strip of paper, which is impregnated with a three per cent. solution of corrosive sublimate containing twelve or fifteen per cent. of iodide of potassium, and also dried. One of each of these strips is shaken up in a glass containing the urine to be tested, and allowed to stand. If albumen be present, the urine at once becomes turbid, and in a short time a flocculent precipitate settles. This reaction is extremely sensitive, and will show the presence of one part of albumen in a thousand. Through the excess of iodide of potassium iodide of mercury and potassium is formed, in the place of the chloride of mercury and potassium, with chloride of potassium in solution. It is through the formation of iodide of potassium and mercury that the albumen is precipitated.

### METHOD FOR THE DETECTION OF SUGAR.

One strip of filter-paper is soaked in a strong solution of pure indigo, and a second in a concentrated solution of bicarbonate of sodium. A piece of the indigo paper is placed in water until it becomes of a light blue color (it must not be too dark). The urine is then added, and the mixture boiled for several minutes with some large pieces of the sodium paper. If sugar be present, the light blue color either disappears or changes into yellow. This test may also be applied to albuminous urine, as the bicarbonate of sodium holds the albumen in solution. It will serve for the detection of sugar in 1-40 or 1-100 per cent. solution.—*St. Louis Medical Journal.*

**A Few Points of Diagnosis.**—J. M. Baker, M.D., in Eclectic Medical Journal, writes:

A doctor's success depends upon his powers of diagnosis; consequently anything to facilitate it is of value. I do not claim the following points to be original with me, but I have observed them.

Large muscles over and just below malar bone indicate good lungs; duskiness or unnatural redness, lung disease.

The vermillion of the lip will show condition of circulation and digestion.

The condition of the liver and portal circle is shown in the end of the nose and the sclerotic coat of the eye; topers are good subjects for observation.

When a patient complains of pain in top of the head, or involuntarily places his hand over the pubic region, look after him with a catheter.

A patient that continually licks his lips while talking to you, has some kidney affection.

A fiery, half-crazy expression of eye, with pimples on forehead and chin, is a sign of self-abuse; a dull, inane, listless, far-away look, impotency and deficient innervation.

Tenderness over second cervical vertebra will confirm diagnosis of pregnancy, in second and third months.

Large parietal eminences and dark complexion generally accompany hysteria.

I give prognosis according to the distance between the mastoid processes, when treating children. A wide or deep base of brain certainly indicates strong viability.

It is generally a good plan to keep your mouth shut and your eyes open, and don't argue with old women about diseases of children.

**The Present Status of the Germ Theory.**—Undoubtedly there are many of our readers, says the New York Medical Record, who are convinced that Klein has dealt a staggering blow to that particular phase of the germ theory which teaches that the comma bacillus is the contagium vivum of Asiatic cholera, and there are many who seem willing to admit that Tait has fairly unhorsed Listerism. This surgeon, with defiant enthusiasm, rejects antisepticism in toto, and, insisting only on the most punctilious cleanliness, sunshine and ventilation, boldly ransacks the abdominal cavity, washing it out with sponges that are scrupulously clean, but not specially asepticized, and undertakes every variety of extirpation with as much confidence as ordinary surgeons amputate an arm or tie an artery.

In this country the weight of evidence, with certainly a great preponderance of authority, is unequivocally in favor of a rational observance of Listerian principles; and, whatever may be the final fate of the germ theory, which many investigators have come to consider as quite positively demonstrated, it is evident that extreme and inflexible views on either side of the question are not in accordance, in its present status, with the true scientific spirit, and not calculated to evolve the truth that must underlie the whole discussion.

**Pilocarpine in Erysipelas.**—Professor Da Costa (College and Clinical Record) thus states the treatment of a case of erysipelas: He was admitted on Thursday morning, with erysipelas of the upper part of the face, which was rapidly spreading over the brow. His pulse was 80; temperature, 102.8°; respiration, 22. The urine was examined, with a negative result. He was ordered tincture of the chloride of iron, 20 gtts. every three hours, but only received on dose; as the disease was rapidly spreading, and something was needed to make a prompt impression, I used another and more active agent. This was not the first case in which I had used this remedy, but it was the first in which I obtained such relief. He received, hypodremically, one-sixth of a grain of the muriate of pilocarpine. The result was remarkable. Here is the temperature record: the temperature fell from 102° to 99½°. He sweated profusely for an hour and a half, and there was no further development of the erysipelas; not only did it not spread further, but what did exist quickly subsided. No local treatment was employed, not even cold applications; therefore, whatever success was obtained was from the pilocarpine.

I call your attention to this treatment of erysipelas. I said that it had not been my first case, although it was the most striking case I have seen. As long as five years ago I used jaborandi in the treatment of erysipelas until sweating was produced, and, I thought, with the result of checking further development. In one case with high temperature the disease had already made some headway, and did not subside so quickly. Under the use of iron the disease had not been controlled, but the fluid extract of jaborandi, given every two hours, checked it. I have since used the jaborandi in connection with the iron, at times, with good results. This is, however, not a new treatment with me, as I have used it for some time. Jaborandi and pilocarpine—its active principle—are, of course, similar in their effects.

I have called your attention to this treatment, not because I believe it will be followed by the same result in every case, but because it is worthy of a trial. If you get a case of erysipelas in its beginning, use pilocarpine. It has saved this man a long and dangerous illness; and, as he had been drinking, as he says he has, the results might have been serious. In the use of this treatment, it should be borne in mind, that, in order to be fully effective, profuse sweating must be produced.

**Rapid Recovery after Wound of Stomach and Protrusion of Viscera.**—A remarkable case of recovery, after severe abdominal injury, is reported in the Gazetta degli Ospitali, by Dr. Iginio Tansini, (Lancet). P. G., aged 46, a peasant of Lodi, was admitted into the local hospital on the 10th of August, in a state of drunkenness, with an extensive wound in the left hypochondriac region. Through it protruded the stomach, the transverse colon, and a large part of the great omentum. On the anterior surface of the stomach was a wound an inch and a quarter long, through which the lining of the mucous membrane bulged so as to fill it. That wound was closed with two fine catgut sutures. After cleaning the displaced viscera with cotton pledgets dipped in a 2½ per cent. solution of carbolic acid, reduction was attempted, but the tightness of the edges prevented it. As the wound reached upwards to the costal margin, it was extended an inch downwards, and the viscera replaced. The parietal wound was then found to measure three and a half inches. A considerable amount of blood having accumulated in the abdominal cavity, the toilette of the peritoneum was very carefully performed with carbolized gauze soaked in warm carbolized water. The first pledgets brought away blood, and others were introduced amongst the coils of intestine and into the pelvis, until they came away quite clean. After inserting a thick drainage-tube into the lower angle of the wound, this was closed with four deep and four superficial sutures of carbolized silk; a broad antiseptic dressing followed. Progress was uninterrupted; no fever supervened. In the first three days much bloody fluid was carried off by the drainage tube, which was gradually shortened, and definitely removed the eighth day. On September 5, the twenty-sixth day after admission, the patient left the hospital perfectly cured.—*Jour. American Med. Association.*

**Health of Children in Los Angeles.**—Dr. Walter Lindley (in the Pacific Medical and Surgical Journal) says: The combined death-rate from the three principal diseases of childhood has been, during the last seven years, less than one per month. It is true the population of Los Angeles has increased, but a glance at the table will show that the death-rate has not increased. The reasons for this light mortality among children in Los Angeles are: 1. The diurnal breeze from the ocean, which constantly purifies the atmosphere; 2. The constant ripening of fruits—oranges and lemons in the winter; apricots, nectarines, peaches, and berries in the spring; apples, pears, and grapes during the summer, and strawberries all the year round; 3. Every variety of vegetables, fresh, each month in the year; 4. The great number of clear days, which, in the language of my friend, Dr. J. P. Widney, "renders possible an out-door life almost every day of the year."

**Kola.**—M. Monnet ("Bull. gen. de therap.") has made an exhaustive study of the physiological and therapeutical properties of this drug. He draws the following conclusions from his experiments: Kola acts as a cardiac stimulant, increasing the number, force, and regularity of the contractions. This property is probably due to the caffeine and theobromine contained in the nut. At a further stage of its action it regulates the pulse, increasing its amplitude while it renders it slower. Since it has a decided diuretic action, kola is especially recommended in cardiac affections accompanied by dropsy. In general, it may be said that the drug increases the contractions of the smooth muscles, while it paralyzes the striated. It has marked tonic properties, and is therefore indicated in anæmia and chronic wasting diseases. Kola favors digestion, either by increasing the secretion of the gastric juice or by causing increased contraction of the smooth muscular fibers. It would seem to be specially indicated in atonic dyspepsia. It is a valuable remedy in chronic diarrhœa, but its exact action in this direction is not known.—*N. Y. Medical Journal.*

**Osmic Acid in Sciatica.**—Mr. James Mercer reports in the Lancet the results of his practice with this drug in eighteen cases, treated at the Bath Mineral Water Hospital. He claims that only about twenty-five per cent. remain of the cases admitted as not benefitted by the waters; and it is to this twenty-five per cent. that he applies this treatment. The patients' ages varied from eighteen to sixty-five. In twelve cases he succeeded in giving them absolute relief for a period of three weeks. The number of injections varied from one to four. In six he gave temporary relief. The injections were as many as twelve in one case, and they obtained more comfort by its use than even by hypodermic injections of morphia. He used a one per cent. solution, injecting deeply over the sciatic nerve, at a point midway between the tuber ischii and trochanter major, three to five minims of the solution. It produced no constitutional effects, but locally at the seat of puncture the patient invariably complained of a numb feeling, which, however, was transient. In some cases the effect was marvellous, the patient being able, after a short time, to stand on the affected side, which he had been unable to do for years.—*Jour. Amer. Med. Assoc'n.*

**Soluble Elastic Capsules of Quinine Sulphate.**—The obstacle to the exhibition of quinine is the bitter taste of the drug. Various devices have, from time to time, been suggested to overcome this obstacle, but the very number of them is a proof of their non-success. When quinine is given in combination with excipients to mask its taste, we have superadded to its medicinal properties those of the excipient, and these are often undesirable. The objections to pills, sugar and gelatin-coated, are also well known. The desideratum in the administration of this drug is a device which is at once easy of deglutition, of perfect solubility in the stomach, and permitting of the uncomplicated action of the salt. This desideratum we have supplied in the soluble elastic capsules of quinine sulphate placed before the public by Messrs. Parke, Davis & Co., of Detroit. In addition to these properties, these capsules are guaranteed to contain the exact amount of quinine specified on the labels, the contents of each one being separately weighed before being encapsuled.—*Ex.*

**Mangifera Indica.**—A writer in Eclectic Medical Journal says of Mango:

"I well remember the first two cases in which I used this remedy. They were the most severe forms of ulcerated sore throat, with considerable constitutional disturbance, in which tinct. ferri. chlor. had been persistently applied for some time without any apparent benefit; but, under the influence of the Mango, in a few hours a change was made for the better. Both the inflammation and ulceration rapidly subsided, and the patient experienced a feeling of relief soon after the first application. This, I may say, has been a very noticeable effect, that the remedy gives, many times, almost immediate relief. It is an especially nice remedy for the sore throats of children; for, when applied with a camel's hair pencil, it is so far from being disagreeable that usually no objection is made to it after the first application.

"In gynæcological cases I find the Mango simply invaluable. In cervicitis with ulceration or erosion of the os, enlargement of the uterine neck, and the cervical canal filled with a transparent, ropy mucus, such as we always find in these cases, I take a pledget of absorbent cotton, around the middle of which a string is tied to facilitate its removal. I saturate it with equal parts of glycerine and specific tincture of Mangifera, place it firmly against the os, and leave it there for twelve or twenty-four hours. Its removal is followed by a gush of watery fluid, due to the action of the glycerine, and in a short time we see the signs of commencing improvement. The cervix becomes paler and of a more healthy color; it decreases in size, and the erosion and leucorrhœal discharge soon disappear. I think my experience in these cases justifies me in condemning the use of caustics, for I believe these agents leave the cervix enlarged and congested—conditions we are trying to remove—and the presence of which are most favorable to a recurrence of the inflammation.

"I have, also, used Mango internally in some cases of menorrhagia, and it has proved quite serviceable; but as my knowledge of its effects from internal administration is comparatively limited, I cannot speak in this respect with as much certainty as I can of its local use."

**Thallin, the New Antipyretic.**—The list of antipyretics has received the principal share of the additions to the materia medica during the past year. Kairine and antipyrine, the chief of these, have excited considerable attention, and have found considerable favor, and now we have still another candidate for a position on the list—thallin. It is a synthetic production, somewhat accidentally discovered by Dr. Von Jaksch, of Vienna, who recently introduced it to the notice of the Society of Physicians of that city. Chemically, thallin is tetrahydroparachinanisol, and we find it difficult to restrain our enthusiasm for the man who gave it forth under its disyllabic synonym. Life is too short, and the danger of sub-maxillary dislocation is too great, to permit the discreet man to employ the chemical name, except on state occasions.

Dr. Von Jaksch maintains that thallin is an antipyretic, pure and simple, in which respect it differs from quinine, salicylic acid, etc., which combine other properties which it is sometimes desirable to avoid. In eighty-six cases, including pneumonia, typhoid fever, erysipelas, measles, etc., in which he employed it, it reduced the temperature with certainty and without any disagreeable or other effects which attend the employment of other antipyretics referred to. In no case had it any effect on the course of the disease, being purely a remedy that reduces temperature. The dose is from four to twelve grains, which is sufficient to keep the temperature down for several hours.—*Med. Age.*

**Gangrene from a Hypodermic Injection of Quinine.**—Dr. F. Tipton, of Selma, Ala., has had a bitter experience with quinine. He had a patient suffering with a violent form of quotidian ague. Nothing would stay on the stomach, or in the rectum, so he injected into the outer aspect of the leg ten grains of quinine dissolved in water with half a grain of tartaric acid to each grain of quinine. After three days, the leg began to swell and became red; then acute spreading gangrene set in. For several days life and limb were threatened, but finally a line of demarcation appeared. The gangrenous area covered a space equal to that of both hands. Dr. Tipton writes that he had seen the injection which he used administered in about one hundred cases with no injury at all. In this case the patient showed a tendency to suppuration, even when he received slight injuries or scratches. Our correspondent is very positive that he will not again run the risk of any such accident as the above.—*N. Y. Med. Rec.*

The case of an old woman, aged seventy, who, after eating a large quantity of watermelon and swallowing the seeds, suffered from obstruction of the bowels, is related by Dr. Ginlio Dozzi in the Gazz. Med. Ital. Purgatives and injections had been tried with no relief. The meteorism was enormous. He determined to try entero puncture, using trocar No. 2 of Dieulafoy's aspirator. Four punctures were made, two in the right iliac region, the third in the left upper fourth, and the fourth in the left lower fourth. From three punctures issued an immense quantity of gas; from the fourth no gas, the trocar being plugged with fæcal matter. A dose of oil given the same evening produced four copious evacuations, and the patient made a good recovery. One of the punctures gave rise to a small abscess. In this case peristaltic action was evidently prevented by the enormous quantity of gas arising from the decomposition of the retained fæces.—*Ibid.*

**Eserine.**—In a paper read before the Midland Med. Society, Mr. Priestley Smith held (British Med. Jour.) that the opposite effects of eserine and atropine in the tension of glaucoma were not due to any influence over secretion, but to changes in the mechanical relations of the iris. Any changes in the tension of the eye which they produced were accomplished by alterating the relations of the iris in such a way as to hinder or promote the escape of the intra-ocular fluid. Clinical experience, and the facts discovered by dissecting in the principal varieties of the disease, supports this idea. As to the propriety of using eserine in any individual case, the question should be asked: Is there an obstruction in the eye which is capable of reduction by contraction of the pupil? Caution was advised in the use of the remedy, as if it did no good it was likely to do harm, for the reason that it increased the amount of blood in the vessels of the iris, and had been known to produce hemorrhage.—*Medical Review.*

**Action of Salicylate of Soda Upon the Uterus.**—The action of salicylate of soda upon the uterus has been carefully studied by M. Balette, in a thesis (Journal de Med. et de Chir. Prat., Nov., 1884) in which he notes some effects that are little known. In dysmenorrhœa, 4 to 6 grammes (ʒj—jss) taken in three doses often calms the pain in a quarter or half an hour, and facilitates the establishment of the flow. This fact had been previously observed by M. Sabalowski.

M. Balette cites several instances of a menorrhagic tendency caused by this drug, which had also been previously observed by M. Bucquoy. He explains this by the fact that this disease has a tendency to cause other forms of hemorrhage as well (hematuria, epistaxis, etc.)

While salicylate of soda has been accused of causing abortions, the results are conflicting. According to M. Balette's experience, this remedy, given in moderate therapeutic doses, is not abortifacient. Still it is necessary to use caution in administering it to pregnant women, as some are predisposed to abortion.—*Weekly Med. Review.*

**Spermatorrhœa—Monobromide of Camphor.**—The monobromide of camphor has been highly lauded for the cure of spermatorrhœa. Dr. L. J. Fogel, calling attention to it, says that he had under his care for the past three months two cases of spermatorrhœa, the subjects being old masturbators, at respectively nineteen and twenty-one years.

Had administered a host of the usual remedies with no satisfactory results; finally, placed them upon the monobromide of camphor, in two to three-grain doses, four times daily, with prompt effect and perfect cures. Deem this remedy as especially adapted to old-standing cases, where the seminal emissions are dependent upon a morbid and relaxed condition of the generative organs.—*Medical Summary.*

**Nitrate of Silver For Fissure of the Anus.**—At a recent meeting of the New York Clinical Society (New York Medical Journal) Dr. Kelsey stated that for the past two years he had not been obliged to stretch the sphincter for fissure of the anus in a single case. He had used instead a weak solution of nitrate of silver, five to ten grains to ounce. In one patient recently under his care a single application of a ten-grain solution effected a cure; another very obstinate case was relieved in three weeks.—*Med. News.*

